# Anti-TNF-*α* Monoclonal Antibody Therapy Improves Anemia through Downregulating Hepatocyte Hepcidin Expression in Inflammatory Bowel Disease

**DOI:** 10.1155/2019/4038619

**Published:** 2019-11-13

**Authors:** Weigang Shu, Zhi Pang, Chunjin Xu, Jian Lin, Gengfeng Li, Wei Wu, Suofeng Sun, Junxiang Li, Xiuling Li, Zhanju Liu

**Affiliations:** ^1^Department of Gastroenterology, Henan Provincial People's Hospital, School of Clinical Medicine, Henan University, Zhengzhou, Henan 450003, China; ^2^Department of Gastroenterology, The Shanghai Tenth People's Hospital of Tongji University, Shanghai 200072, China; ^3^Department of Gastroenterology, Suzhou Municipal Hospital Affiliated to Nanjing Medical University, Suzhou 215008, China; ^4^Department of Gastroenterology, Shangqiu City First People's Hospital of Xinxiang Medical University, Shangqiu 476100, China; ^5^Department of Gastroenterology, Dongfang Hospital, Beijing University of Chinese Medicine, Beijing 100078, China

## Abstract

Anemia is one of the most common complications in patients with inflammatory bowel disease (IBD). Hepcidin as a key regulator of iron metabolism is pivotal in mediating the occurrence of anemia of chronic disease. Herein, we analyzed the levels of hepcidin in sera from IBD patients by enzyme-linked immunosorbent assay and investigated its potential role in regulating the anemia in IBD. We observed that the levels of serum hepcidin were increased in active IBD patients compared with those in remitted IBD patients and healthy controls and that serum hepcidin was associated with disease activity, CRP, and ESR, respectively. Importantly, we found that the increased levels of serum hepcidin were positively correlated with the severity of anemia and the imbalance of iron metabolism in anemic UC and CD patients. Proinflammatory factors (e.g., IL-6, IL-17, and TNF-*α*) were positively correlated with the concentrations of serum hepcidin in IBD patients. Interestingly, hepcidin was found to be decreased in patients with Crohn's disease after successful therapy with anti-TNF-*α* mAb (i.e., infliximab), indicating the underlying association between TNF-*α* and hepcidin expression. To investigate the specific mechanisms involved, we cultured LO2 and HepG2 cell lines *in vitro* under stimulation with TNF-*α* and observed that the levels of hepcidin mRNA were markedly upregulated in caspase-3/8- and NF-*κ*B-dependent manners. Therefore, our data suggest that TNF-*α* stimulates the expression of hepcidin in IBD patients, resulting in aggravated anemia and that blockage of TNF-*α* or the caspase-3/8 and NF-*κ*B pathways could downregulate hepcidin expression. This study provides inspiration for the therapy and management of anemia in IBD.

## 1. Introduction

Inflammatory bowel disease (IBD) is a group of chronic inflammatory diseases caused by dysregulated immune response to microbiota in the gastrointestinal tract and consists of two major forms, including Crohn's disease (CD) and ulcerative colitis (UC). Abdominal pain, diarrhea, weight reduction, and chronic fatigue are the main clinical characteristics of IBD, which significantly impair the quality of patients' life. Recent data have demonstrated that the chronic fatigue of patients is often closely associated with anemia [1, 2]. Although often neglected, anemia is known as the most frequent comorbidity of IBD [3], which may progress in the course of inflammatory response, intestinal bleeding, disorders of iron absorption [4, 5], vitamin B12 and folate deficiency, drug-related side effects, and hematologic diseases [6–8]. Previous study has demonstrated that the prevalence of anemia in patients with IBD in European countries is 21% to 27% and that 57% of anemic patients with IBD are iron deficient [9], emphasizing the importance to the maintenance of iron metabolism balance in clinical treatment.

Hepcidin, a cysteine-rich and antimicrobial peptide, is mainly synthesized and secreted by the liver. It consists of 8 cysteine residues, forming a single hairpin structure which contains 4 two-sulfur bonds, and its amino acid sequence is highly conserved among different mammals [10, 11]. Currently, three types of hepcidin have been found, including Hepc22, Hepc25, and Hepc20, and the latter two are regarded as the main existing forms. Human hepcidin has a wide range of antimicrobial and antiprotozoan functions [12, 13]. Injury, infection, and inflammatory response are found to strongly increase hepcidin expression, and hepcidin is an important effector molecule affecting iron transport between different iron pools. Moreover, hepcidin is responsible for the regulation of the iron homeostasis in the human body and other mammals by binding and downregulating ferroportin (FPN), the only known iron exporter that promotes iron absorption by duodenal enterocytes and pumps iron out of phagocytes and hepatocytes [14–16]. Therefore, the hepcidin-FPN axis is considered as the central player in regulating iron homeostasis [17].

The inflammatory response has been found to be involved in iron metabolism in the human body, and the discovery of hepcidin provides a way to explain the relationship between immune response and iron metabolism. To date, IL-6 is found to increase the expression of hepcidin, while the expression of hepcidin in IL-6 knockout mice does not change much even with the injection of endotoxin [18, 19]. Tumor necrosis factor- (TNF-) *α*, produced as a soluble or transmembrane protein, is one of the key proinflammatory factors in immune response. After binding to receptors TNFR1 and TNFR2, NF-*κ*B is activated and various biologic functions are conducted subsequently [20]. Previous work has demonstrated that TNF-*α* induces the anemia in IBD patients by weakening absorption of iron [21, 22], while anti-TNF-*α* therapy improves anemia in CD patients and is associated with the decreased levels of serum hepcidin [23, 24]. However, whether TNF-*α* directly stimulates hepcidin expression and the mechanisms involved are still unclear.

In this study, we investigated hepcidin expression in the sera of IBD patients and found that the concentrations of hepcidin were higher in the sera of active IBD patients than in remitted IBD patients and healthy controls. The levels of hepcidin were also significantly increased in anemic UC and CD patients than in nonanemic patients, which were positively correlated with the severity of anemia and the imbalance of iron metabolism, and relevant to disease activity, CRP, and ESR of IBD patients. Moreover, the levels of hepcidin were associated with the levels of proinflammatory cytokines (e.g., TNF-*α*, IL-6, and IL-17) in IBD patients. Interestingly, the application of anti-TNF-*α* mAb could effectively suppress hepcidin expression in active CD patients and significantly improve the status of anemia. *In vitro* experiments were also conducted to reveal that TNF-*α* could enhance the expression of hepcidin in both LO2 cells and HepG2 cells in caspase 3/8- and NF-*κ*B-dependent manners. Therefore, our study suggests that hepcidin is increased in active IBD patients and that TNF-*α* could facilitate hepatocytes to produce hepcidin during inflammatory response in IBD. Our study highlights that the application of anti-TNF-*α* mAb or inhibitors of caspase 3/8 and NF-*κ*B could reverse the increase of hepcidin, which may be effective for the treatment of chronic inflammatory anemia caused by long-term hepcidin overexpression.

## 2. Materials and Methods

### 2.1. Ethical Considerations

This study was approved by the Review Boards for Clinical Research from both Henan Provincial People's Hospital and Shanghai Tenth People's Hospital. The methods were conducted according to the approved guidelines, and informed permission was also obtained from all subjects.

### 2.2. Patients and Sample Collection

Information of all patients with IBD was collected from the Department of Gastroenterology of Shanghai Tenth People's Hospital from January 2017 to December 2018. The combination of conventional clinical symptoms and radiological and endoscopic features contributed to establish the diagnosis of UC and CD. 66 patients with CD (25 active CD patients and 41 CD patients in remission), 33 patients with UC (22 active UC patients and 11 UC patients in remission), and 22 healthy volunteers were recruited to donate serum samples. Glucocorticoids, iron supplementary therapy, biological agents, and blood transfusion therapy had not been given to all patients in the last one month when recruited, and all patients had no other autoimmune diseases like rheumatoid arthritis and autoimmune hepatitis and infectious diseases like intestinal tuberculosis and intestinal amebic dysentery infection. The clinical characteristics of patients and healthy volunteers are described in Table 1.

### 2.3. Analysis of Routine Biochemical Parameters

EDTA anticoagulated blood samples were obtained from both IBD patients and healthy volunteers after overnight fasting. Hemoglobin (Hb), mean corpuscular volume (MCV), mean corpuscular hemoglobin (MCH), mean corpuscular hemoglobin concentration (MCHC), ferritin, vitamin B12, folic acid, erythrocyte sedimentation rate (ESR), and C-reactive protein (CRP) were measured on the Beckman LX20 analyzer (Brea, CA, USA) according to routine laboratory tests.

### 2.4. Reagents

The ELISA kit for analysis of human hepcidin was purchased from CUSABIO (College Park, MD, USA). ELISA kits for human TNF-*α*, IL-17, and IFN-*γ* were all purchased from BioLegend (San Diego, CA, USA). The RNeasy kit was purchased from Qiagen (Valencia, CA, USA). SYBR PrimeScript RT reagent kits were purchased from TaKaRa (Dalian, China). Dulbecco's Modified Eagle's Medium (DMEM), fetal bovine serum (FBS), penicillin (100 U/mL) and streptomycin (100 g/mL), L-gentamycin, and 2-ME were all purchased from HyClone (Logan, UT, USA). Human normal LO2 hepatocytes and human liver-derived hepatoma G2 cells (HepG2) were purchased from the Chinese Academy of Sciences Committee Type Culture Collection cell bank (Shanghai, China). The CCK-8 kit was purchased from the Shanghai Yeasun Biotechnology Company, Ltd. (Shanghai, China). The JNK inhibitor (JNK-IN-8, 10 *μ*M), NF-*κ*B inhibitor (BAY 11-7082, 10 *μ*M), and caspase-3/8 inhibitor (Z-DEVD-FMK, 50 *μ*M) were purchased from Selleckchem (Shanghai, China).

### 2.5. Enzyme-Linked Immunosorbent Assay (ELISA)

Blood samples were allowed to clot for 2 h at room temperature or overnight at 4°C before centrifugation for 15 min at 1000 g. The serum was then removed immediately and stored at -80°C for further analysis.

### 2.6. Culture of LO2 Cells and HepG2 Cells

DMEM supplemented with 10% heat-inactivated fetal bovine serum (FBS), penicillin (100 U/mL), and streptomycin (100 g/mL) was used to culture human normal hepatocyte LO2 cell line and human liver-derived hepatoma G2 cell line (HepG2). TNF-*α* (10 ng/mL), IL-6 (10 ng/mL), and LPS (100 ng/mL) were used to stimulate these cell lines, respectively, and DMEM supplemented with 2% heat-inactivated FBS, penicillin (100 U/mL), and streptomycin (100 g/mL) was used during treatment. After 6, 12, and 24 h of culture, cells were harvested and the total RNA was extracted using the RNeasy kit according to the manufacturer's instructions. The mRNA levels of hepcidin were analyzed by qRT-PCR. To further investigate the mechanism whereby TNF-*α* regulates hepcidin expression, anti-TNF-*α* mAb (infliximab, IFX, 50 ng/*μ*L), JNK inhibitor (JNK-IN-8, 10 *μ*M), NF-*κ*B inhibitor (BAY 11-7082, 10 *μ*M), and caspase-3/8 inhibitor (Z-DEVD-FMK, 50 *μ*M) were added into the culture. After 12 h of culture, cells were harvested and the expression of hepcidin mRNA was detected by qRT-RCR.

### 2.7. Cell Viability Assay

The CCK-8 kit was used to determine cell viability and the optical density value (OD value) which was in direct proportion to the number of living cells was used to express cell viability. A microplate reader was used to measure the absorbance at 450 nm. All experiments were performed in triplicate.

### 2.8. Quantitative Real-Time Polymerase Chain Reaction (qRT-PCR)

The RNeasy kit was used to extract the total RNA in accordance with the manufacturer's instructions. A NanoVue spectrophotometer (GE Healthcare; Piscataway, NJ, USA) was used to assess the quantity and quality of the samples, with 280/260 > 1.8 and 28S/18S > 1.4. The TaKaRa SYBR PrimeScript reverse RT reagent kit was used to synthesize the complementary, conforming to the manufacturer's instructions. Reverse transcription PCR reactions were conducted under the following conditions: 25°C for 10 min, followed by 42°C for 15 min and 85°C for 5 min. SYBR green methodology was used to perform quantitative real-time PCR (qRT-PCR) in the ABI prism 7900 HT sequence detector (Applied Biosystems; Foster City, CA, USA). GAPDH was analyzed as the endogenous reference gene. The syntheses of all primers were conducted by ShengGong Biotech (Shanghai, China) according to standard methodologies. PCR reactions were conducted with 40 cycles under the setting conditions: 95°C for 1 min, followed by 40 cycles at 95°C for 15 sand 60°C for 30 s. Triplicate wells were used to perform qRT-PCR analysis for all samples. The relative levels of target gene expression were analyzed as a ratio relative to the GAPDH reference mRNA. The specific primers were synthesized as follows: hepcidin (sense, 5′-CTGACCAGTGGCTCTGTTTTC-3′; antisense, 5′-GAAGTGGGTGTCTCGCCTC-3′) (ShengGong Biotech; Shanghai, China).

### 2.9. Anti-TNF-*α* mAb Treatment in Patients with Crohn's Disease

Patients with active Crohn's disease (A-CD, *n* = 32) were recruited from the Department of Gastroenterology of Shanghai Tenth People's Hospital and received iv injection of anti-TNF-*α* mAb (i.e., infliximab, IFX) at the dose of 5 mg/kg (Cilag AG; Schaffhausen, Switzerland) at weeks 0, 2, and 6 as described previously [22, 25]. The characteristics of CD patients including age, sex, smoking history, medical treatment, disease duration, and lesion areas are described in Table 2. The clinical response in these patients was recorded weekly, and CD patients were classified into two groups according to the changes of Crohn's disease activity index (CDAI), including the Response group (CDAI < 150 or decrease of CDAI score ≥ 70 points) and the Failure group (CDAI > 150 and decrease change of the CDAI < 70 points). Serum samples were collected prior to and 12 weeks after the first anti-TNF-*α* mAb therapy, and the levels of hepcidin were analyzed by an ELISA.

### 2.10. Statistical Analyses

All data were expressed as mean ± standard error of the mean (S.E.M.). GraphPad Prism 6 and SPSS statistics version 14.0 (SPSS; Chicago, IL, USA) were used to perform data analysis. Unpaired Student's *t*-test and one-way analysis of variance (ANOVA) were used to calculate statistical significance. Spearman's correlation was used to perform correlation analysis. A value of *P* < 0.05 was considered statistically significant.

## 3. Results

### 3.1. Hepcidin Increases in the Sera of IBD Patients and Is Relevant to Disease Activity, CRP, and ESR, Respectively

**Figure 1:**
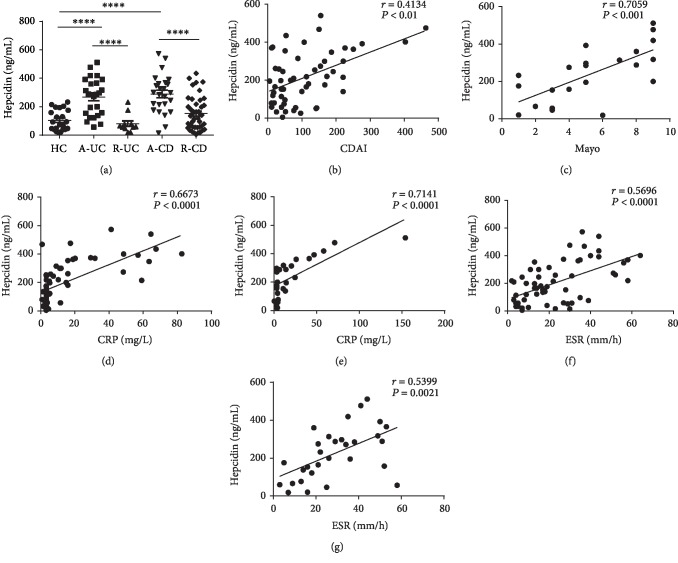
Hepcidin is increased in the sera of IBD patients and associated with disease activity, C-reactive protein (CRP), and erythrocyte sedimentation rate (ESR), respectively. (a) Serum samples were collected from healthy control (HC, *n* = 22), active CD patients (A-CD, *n* = 25), CD patients in remission (R-CD, *n* = 41), active UC patients (A-UC, *n* = 22), and UC patients in remission (R-UC, *n* = 11), and hepcidin was analyzed by ELISA. ^∗∗∗∗^*P* < 0.0001, ^∗∗∗^*P* < 0.001. Data were expressed as mean ± SEM, unpaired Student's *t*-tests. (b) Correlation between Crohn's disease activity index (CDAI) and hepcidin in patients with CD (*n* = 54). (c) Correlation between Mayo score and hepcidin in patients with UC (*n* = 22). (d) Correlation between CRP and hepcidin in patients with (*n* = 60). (e) Correlation between CRP and hepcidin in patients with UC (*n* = 29). (f) Correlation between ESR and hepcidin in patients with CD (*n* = 60). (g) Correlation between ESR and hepcidin in patients with UC (*n* = 30). Correlation analysis was performed by Spearman's correlation analysis. *P* values are shown in each panel.

Our previous study has demonstrated that the incidence of anemia is increased in IBD patients, especially in patients with active disease [22]. To further investigate the clinical relevance of anemia to the pathogenesis of IBD, we focused on hepcidin which plays a pivotal role in regulating iron metabolism. We defined CDAI ≥ 150 as active CD (A-CD), CDAI < 150 as CD in remission (R-CD), Mayo score ≥ 3 as active UC (A-UC), and Mayo score ≤ 2 as UC in remission (R-UC) in our study. By measuring the serum concentration of hepcidin by ELISA, we observed that the concentrations of hepcidin were higher in the sera of active IBD patients compared with those in remitted IBD patients (*P* < 0.0001 in A-UC vs. R-UC and *P* < 0.0001 in A-CD vs. R-CD) and healthy controls (HC) (*P* < 0.0001 in A-UC vs. HC, and *P* < 0.0001 in A-CD vs HC), while no difference in serum hepcidin was detected between IBD patients in remission stage and healthy controls (Figure 1(a)).

A positive correlation was displayed between CDAI and serum hepcidin in CD patients (*r* = 0.4134, *P* < 0.01) as well as the Mayo score and serum hepcidin in UC patients (*r* = 0.7059, *P* < 0.001) (Figures 1(b) and 1(c)). CRP and ESR, which are routinely used for evaluation of the disease activity in IBD, were also analyzed in these patients, and serum hepcidin was identified to be positively correlated with CRP (*r* = 0.7141, *P* < 0.0001 in UC; *r* = 0.6673, *P* < 0.0001 in CD) and ESR (*r* = 0.5399, *P* = 0.0021 in UC; *r* = 0.5696, *P* < 0.0001 in CD) in IBD patients, respectively (Figures 1(d)–1(g)). Collectively, these data illustrate that hepcidin is significantly increased in active IBD patients in comparation with that in remitted IBD patients and healthy controls and that hepcidin in IBD patients is relevant to disease activity, CRP, and ESR, respectively.

### 3.2. The Levels of Hepcidin Is Associated with the Severity of Anemia in IBD Patients

**Figure 2:**
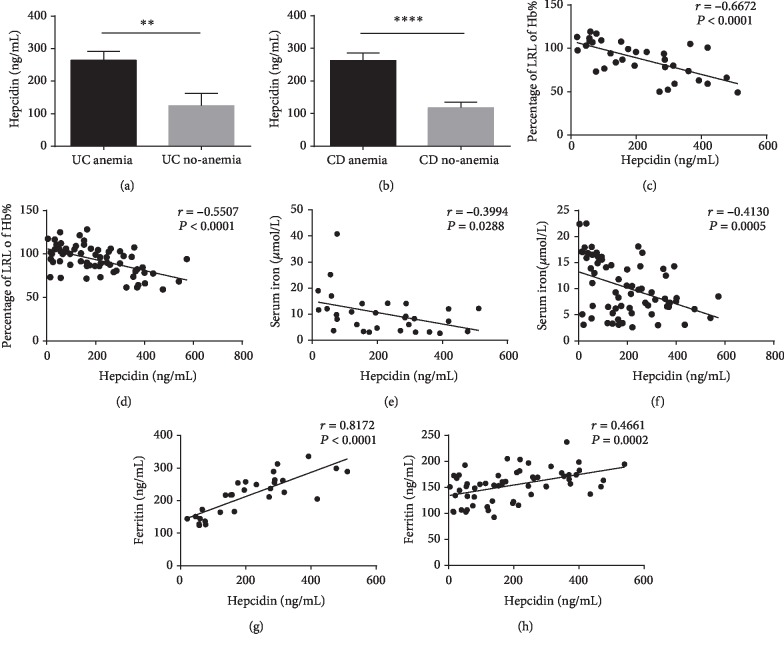
The serum levels of hepcidin are correlated with the severity of anemia in IBD patients. (a) The serum levels of hepcidin in UC patients with anemia (*n* = 21) and UC patients without anemia (*n* = 12). (b) The serum levels of hepcidin in CD patients with anemia (*n* = 36) and CD patients without anemia (*n* = 28). ^∗∗∗∗^*P* < 0.0001, ^∗∗∗^*P* < 0.001, and ^∗∗^*P* < 0.01. Data were expressed as mean ± SEM, unpaired Student's *t*-tests. (c) Correlation between hepcidin and percents of LRL of Hb in UC patients (*n* = 33). (d) Correlation between hepcidin and percentages of LRL of Hb in CD patients (*n* = 66). (e) Correlation between hepcidin and serum iron in UC patients (*n* = 30). (f) Correlation between hepcidin and serum iron in CD patients (*n* = 66). (g) Correlation between hepcidin and serum ferritin in UC patients (*n* = 33). (h) Correlation between hepcidin and serum ferritin in CD patients (*n* = 60). Correlation analysis was performed by Spearman's correlation analysis. *P* values are shown in each panel.

As we demonstrated above, the concentrations of hepcidin were significantly increased in the sera of active IBD patients in comparation with those IBD patients in remission and healthy controls, while whether the distinct hepcidin expression affected the occurrence of anemia or not was still unclear. To further study the relationship between hepcidin and anemia in IBD patients, we defined the Hb level less than 130.0 g/L in men and less than 120.0 g/L in nonpregnant women as anemia [26]. The ratio of Hb of patients when recruited at the study to the lower range limit (LRL) was used to appraise the degree of anemia. For intuitive analysis, we arbitrarily set the level of Hb at the LRL to 100%, and the percentage was used to express the relative changes of Hb levels in IBD compared with the LRL. In our study, 59 patients (21 UC and 38 CD) were found to be anemic, and the incidence of anemia was 63.64% in UC and 57.58% in CD, respectively. In addition, the serum levels of hepcidin were observed to be higher in UC and CD patients with anemia than in UC and CD patients without anemia (*P* < 0.01 in UC; *P* < 0.0001 in CD) (Figures 2(a) and 2(b)). Negative correlations were disclosed between serum hepcidin and the percentages of LRL of Hb in UC patients (*r* = −0.6672, *P* < 0.0001) as well as serum hepcidin and the percentages of LRL of Hb in CD patients (*r* = −0.5507, *P* < 0.0001) (Figures 2(c) and 2(d)). Therefore, we speculated that the increased expression of hepcidin exacerbated the severity of anemia in active IBD patients, but how hepcidin influenced the occurrence of anemia was still not understood. For this reason, the concentrations of serum iron, ferritin, folate, and vitamin B12 were also analyzed in IBD patients. As shown in Figures 2(e)–2(h), negative correlations were illustrated between serum hepcidin and iron in both UC and CD patients (*r* = −0.3994, *P* = 0.0288 in UC; *r* = −0.4130, *P* = 0.0005 in CD), while positive correlations were observed between serum hepcidin and ferritin (*r* = 0.8172, *P* < 0.0001 in UC; *r* = 0.4661, *P* = 0.0002 in CD). However, there were no associations existing between serum hepcidin and folate and vitamin B12 (supplementary Figures [Supplementary-material supplementary-material-1]). These results indicate that hepcidin aggravates the severity of anemia in IBD patients by spoiling the balance of iron metabolism.

### 3.3. Relevance of Biochemical Parameters to the Anemia of IBD Patients

As shown in Table 3, the levels of mean corpuscular volume (MCV), mean hemoglobin content (MCH), and mean corpuscular hemoglobin concentration (MCHC) obviously decreased in CD patients with anemia compared with those in CD patients without anemia (*P* < 0.0001). The levels of MCV, MCH, and MCHC were also observed lower in UC patients with anemia than that in UC patients without anemia (*P* < 0.0001 for MCV, *P* < 0.0005 for MCH and MCHC). These results demonstrated that IBD patients in our study had microcytic hypochromic anemia, which mainly consists of iron-deficiency anemia (IDA) and anemia of chronic disease (ACD). Moreover, we found that the levels of serum iron in UC and CD patients with anemia were much lower than those in UC and CD patients without anemia (*P* < 0.0001 in UC, *P* < 0.0001 in CD). However, no significant differences in serum ferritin, vitamin B12, and folic acid were observed between anemia and nonanemia groups. Taken together, these results indicate that anemia frequently occurs in IBD patients, which is mainly attributed to abnormal iron metabolism.

### 3.4. Correlations between Serum Hepcidin and Proinflammatory Factors

**Figure 3:**
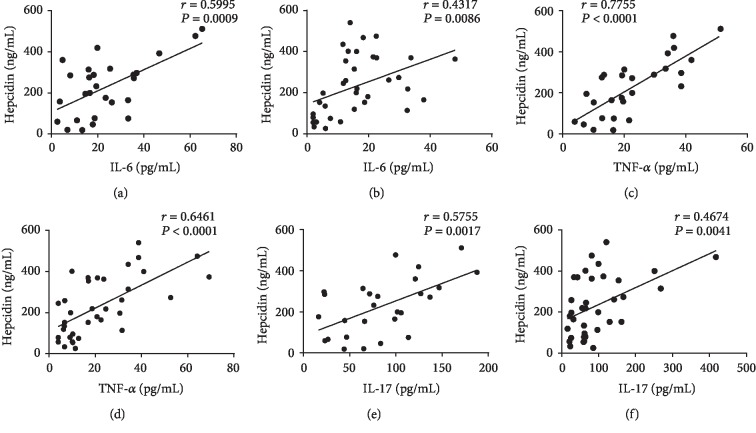
The levels of hepcidin are relevant to IL-6, TNF-*α*, and IL-17, respectively, in the sera of IBD patients. (a) Correlation between the levels of hepcidin and IL-6 in UC patients (*n* = 27), (b) correlation between the levels of hepcidin and IL-6 in CD patients (*n* = 36), (c) correlation between the levels of hepcidin and TNF-*α* in UC patients (*n* = 27), (d) correlation between the levels of hepcidin and TNF-*α* in CD patients (*n* = 36), (e) correlation between the levels of hepcidin and IL-17 in UC patients (*n* = 27), and (f) correlation between the levels of hepcidin and IL-17 in CD patients (*n* = 36). Correlation analysis was performed by Spearman's correlation analysis. *P* values are shown in each panel.

Alternative relapsing and remitting inflammation in intestinal mucosa are frequently present in IBD patients, and considerable amount of proinflammatory factors (e.g., TNF-*α*, IFN-*γ*, IL-6, and IL-17) in intestinal microenvironment plays important roles in impairing iron absorption and erythropoietin production in response to anemia. Moreover, the increased hepcidin expression is also attached to the inflammatory response to some degree. Although previous study [27] has proved that IL-6 is the main factor stimulating the expression of hepcidin through the STAT3 signaling pathway in patients with septicemia and burns and that the high level of hepcidin mRNA is also observed in IL-6-deficient mice with chronic inflammation [28]. We asked whether there were one or more kinds of other inflammatory factors existing in the microenvironment which have the same compacts on hepcidin as IL-6. Thus, in our study, the serum concentrations of IL-6, TNF-*α*, IFN-*γ*, and IL-17 were detected and analyzed. As shown in Figures 3(a)–3(f), the concentrations of serum IL-6, TNF-*α*, and IL-17 were observed to be positively correlated to serum hepcidin in both UC and CD patients, respectively (*r* = 0.5995, *P* = 0.009 for IL-6 in UC; *r* = 0.4317, *P* = 0.0086for IL-6 in CD; *r* = 0.7755, *P* < 0.0001 for TNF-*α* in UC; *r* = 0.6461, *P* < 0.0001 for TNF-*α* in CD; *r* = 0.5755, *P* = 0.0017 for IL-17 in UC; and *r* = 0.4674, *P* = 0.0041 for IL-17 in CD). However, no significant association was observed between serum IFN-*γ* and hepcidin. These data imply that the high levels of inflammatory factors like TNF-*α* and IL-17 facilitate the expression of hepcidin through some underlying regulatory mechanisms, further aggravating anemia of IBD patients.

### 3.5. Anti-TNF-*α* mAb Therapy Downregulates Hepcidin Expression in CD Patients

**Figure 4:**
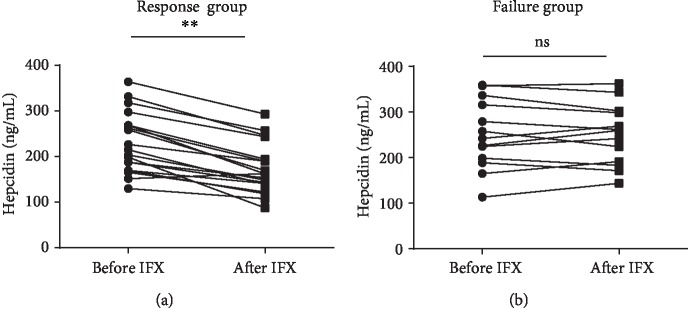
Anti-TNF-*α* mAb therapy downregulates hepcidin expression in CD patients. 32 patients with A-CD were treated with anti-TNF-*α* mAb (infliximab, IFX) at weeks 0, 2, and 6. Serum samples were collected from these patients prior to and 12 weeks after the first anti-TNF-*α* mAb therapy; the levels of hepcidin in the Response group ((a), *n* = 19) and the Failure group ((b), *n* = 13) were analyzed by an ELISA. ^∗∗^*P* < 0.01. Unpaired Student's *t*-tests were performed for statistical analysis.

After confirming the positive correlation between TNF-*α* and hepcidin expression in IBD patients, we then speculated whether the clinical application of IFX for IBD patients could downregulate hepcidin expression. 32 patients with active CD were selected and treated with anti-TNF-*α* mAb (infliximab, IFX) at the dose of 5 mg/kg at weeks 0, 2, and 6 according to the protocol as described previously [25]. The clinical response in these patients was recorded weekly, and they were classified into two groups according to the changes of CDAI, including the Response group (CDAI < 150 or decrease of CDAI score ≥ 70 points) and the Failure group (CDAI > 150 and decrease change of the CDAI < 70 points). Serum samples were collected prior to and 12 weeks after the first anti-TNF-*α* mAb therapy, and the levels of hepcidin were analyzed by an ELISA. The changes of a CDAI score manifested that 19 patients (59.37%) were responsive to IFX therapy and 13 patients (40.63%) failed to IFX therapy. As shown in Figure 4, the expression of hepcidin in serum samples was significantly decreased at week 12 after patients accepted the first IFX infusion compared with that prior to IFX treatment in the Response group (*P* < 0.01 in the Response group), while such change was not observed in the Failure group.

### 3.6. TNF-*α* Upregulates Hepcidin Expression in LO2 Hepatocyte and HepG2 in Caspase 3/8- and NF-*κ*B-Dependent Manners

**Figure 5:**
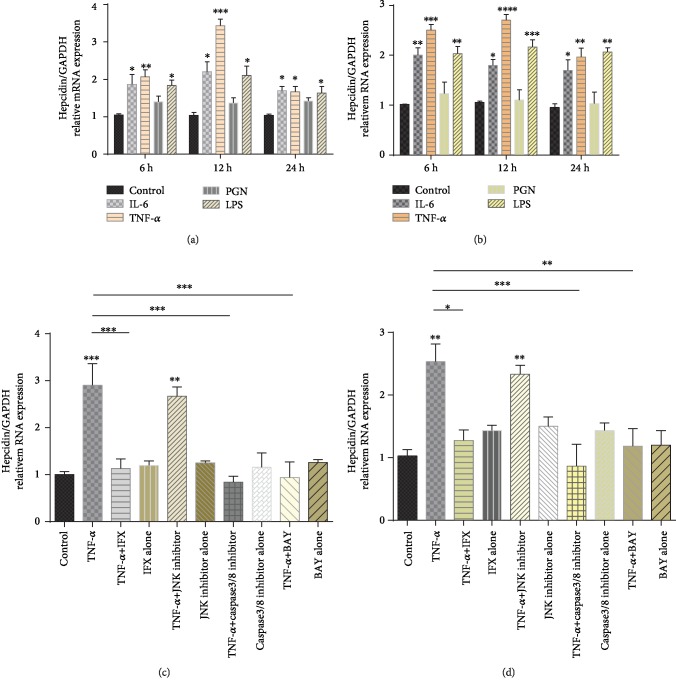
TNF-*α* upregulates hepcidin expression in liver cell line in caspase 3/8- and NF-*κ*B-dependent manners. (a) LO2 cell line and (b) HepG2 cell line were cultured *in vitro* and stimulated with IL-6 (10 ng/mL), TNF-*α* (10 ng/mL), PGN (100 ng/mL), and LPS (100 ng/mL), respectively. After 6, 12, and 24 h of culture, cells were collected and the mRNA levels of hepcidin were determined by qRT-PCR. One-way ANOVA was performed for statistical analysis. ^∗^*P* < 0.05, ^∗∗^*P* < 0.01, ^∗∗∗^*P* < 0.001, and ^∗∗∗∗^*P* < 0.0001 versus controls. (c) LO2 cell line and (d) HepG2 cell line were incubated with TNF-*α* (10 ng/mL) in the presence of anti-TNF-*α* mAb (IFX, 50 ng/*μ*L), JNK inhibitor (JNK-IN-8, 10 *μ*M), NF-*κ*B inhibitor (BAY 11-7082, 10 *μ*M), and caspase-3/8 inhibitor (Z-DEVD-FMK, 50 *μ*M), respectively, as indicated. After 12 h of culture, cells were collected and qRT-PCR was used to determine mRNA levels of hepcidin expression. ^∗∗^*P* < 0.01, ^∗∗∗^*P* < 0.001. GAPDH mRNA levels in each sample were used to normalize gene expression, and the graph represents the combined data of 3 independent experiment (*N* = 3) done each one in triplicate.

Since our previous study highlighted the close association between TNF-*α* and hepcidin, and since the suppressed hepcidin expression observed in CD patients who were responsible to IFX treatment could be attributed to the antagonism of TNF-*α*, we then hypothesized that proinflammatory factors like TNF-*α* may also stimulate the expression of hepcidin in IBD patients. To further disclose the specific mechanism involved, the liver cell lines (i.e., LO2 and HepG2) were cultured *in vitro* under stimulation with LPS, PGN, TNF-*α*, and IL-6, respectively, mimicking to stimulate the activity of human hepatocytes in inflammatory microenvironment. LPS or PGN was also used in our experiment as an efficient inducer of inflammatory response *in vitro*. The expression of hepcidin in LO2 cells and HepG2 cells in the presence of LPS, TNF-*α*, and IL-6 was found to be significantly increased than that of the control group after 6, 12, and 24 h of culture (Figures 5(a) and 5(b)), while PGN did not show such a change. TNF-*α* has been proven to play an essential role in the pathogenesis of IBD as a major proinflammatory factor of apoptosis and proliferation, and the clinical application of an anti-TNF-*α* agent is reported to achieve positive influence in the management of anemia in IBD patients, whereas recognition of the specific mechanism is still limited [29]. Thus, TNF-*α* was selected to further determine the underlying mechanisms involved in the regulation of hepcidin expression in our study. An antibody directed against TNF-*α* (IFX) and JNK inhibitor (JNK-IN-8), NF-*κ*B inhibitor (BAY 11-7802), and caspase-3/8 inhibitor (Z-DEVD-FMK) was adopted and coincubated with LO2 cells and HepG2 cells, respectively. As shown in Figures 5(c) and 5(d), TNF-*α* significantly upregulated the expression of hepcidin mRNA after 12 h of culture. Moreover, hepcidin expression was significantly suppressed in the coincubation with anti-TNF-*α* mAb, caspase-3/8 inhibitor, and NF-*κ*B inhibitor, respectively, compared with TNF-*α* alone, while such reversion was not observed in the JNK signaling inhibitor- (JNK-IN-8-) treated group. Moreover, no significant differences in the viability of LO2 cells and HepG2 cells were observed after exposure to TNF-*α*, IFX, and JNK-IN-8, BAY 11-7802, and Z-DEVD-FMK, respectively, for 12 h (Supplementary [Supplementary-material supplementary-material-1]). Accordingly, our data demonstrate that the treatment of anti-TNF-*α* antibody, caspase-3/8, or NF-*κ*B inhibitor markedly suppresses the upregulation of hepcidin mRNA expression by TNF-*α* stimulation.

## 4. Discussion

Anemia is considered as one of the most common complications in the clinical course of IBD patients, which often causes weakened capacity of physical performance, mood, and cognitive function, leading to poor quality of life. Anemia in IBD is often caused by chronic inflammation of gastrointestinal mucosa, impaired absorption of folate, or vitamin B12, deficiency of iron, and chronic intestinal blood loss. In addition, it is reported that more than half cases of anemia are the results of iron deficiency, emphasizing the importance of iron supplement in the treatment of anemia. The average iron store of adult maintains at least 3-4 g [30], and the body iron homeostasis is balanced between dietary intake and physiologic loss. For IBD patients, there are considerable amounts of factors for shortage of iron, including an increase of iron loss from bleeding as a consequence of gastrointestinal inflammation and weakened iron absorption due to short bowl syndrome, lack of appetite, blockage of intestinal iron acquisition, and reutilization of macrophage as a result of inflammation. In our study, 63.64% UC and 57.58% CD patients were anemic, and the incidence of anemia was significantly higher than that of resident Chinese reported in a nationwide survey before [31]. And the levels of MCV, MCH, and MCHC were much lower in UC and CD patients with anemia than that in UC and CD patients without anemia, but significant differences were not observed in folate and vitamin B12. These results indicate that microcytic hypochromic anemia, mainly composed of IDA and ACD, is involved in the occurrence of anemia. Although both IDA and ACD are reported to be the common sources of anemia, accumulating evidence has claimed that ACD is relatively more frequent to be diagnosed compared with IDA in IBD patients. The pathogenesis of ACD is closely related to the dysregulated immune response in microenvironment, including an increase of proinflammatory factors [32], and the level of serum ferritin has a high accuracy in the diagnosis of simple IDA and can effectively differentiate ACD from IDA. Serum ferritin decreases significantly in patients with IDA, but it does not decrease or even increases in patients with ACD. Moreover, in our study, no significant decrease in serum ferritin was observed in UC and CD patients with anemia compared with that in UC and CD patients without anemia. Taken together, our results suggest that the occurrence of anemia in IBD patients mainly results from ACD, with disordered iron metabolism.

Our previous work [24] have built a link between inflammation and disordered iron metabolism, and we have verified that divalent metal-ion transporter 1 (DMT-1) is decreased in intestinal epithelial cells and contributes to the anemia in IBD. The maintenance of iron homeostasis in physiological state requires the participation of many factors such as DMT-1, ferroportin, and hepcidin. Hepcidin, a liver-derived small peptide with antimicrobial activity, is normally expressed by the liver in physiological state and mainly distributed in the blood and eliminated via the kidneys [33]. In early studies [34, 35], hepcidin is considered as a marker of inflammatory factors which is tightly associated with disordered immunity, and nowadays, it has been implicated in the development of inflammatory-associated anemia (IAA) and ACD [36]. The increased serum hepcidin is often observed in ACD, while the decreased serum hepcidin is the cue of IDA. In addition, hepcidin also plays an essential role in iron regulation and functions as the vital inhibitor which hampers the duodenum to absorb iron and downregulates the iron release from macrophages. A recent work [37] has shown that hepatocytes increase the expression and synthesis of hepcidin under the conditions of chronic inflammation or iron overload and that the use of inhibitor to hepcidin leads to better management of the iron imbalance and ACD. In our study, the concentration of serum hepcidin was significantly increased in active IBD patients in comparation with that in remitted IBD patients and healthy controls, and serum concentration of hepcidin in IBD patients was relevant to disease activity, CRP, and ESR, respectively. Therefore, it can be inferred that the increased disease activity stimulates hepcidin expression. Consistently, our study illustrates that the serum hepcidin is higher in anemic IBD patients compared with IBD patients without anemia, further supporting that the ACD is mainly involved in anemia in IBD. Negative correlations were disclosed between serum hepcidin and percentages of LRL of Hb in UC patients as well as serum hepcidin and percents of LRL of Hb in CD patients. Moreover, negative correlations were also illustrated between serum hepcidin and serum iron in both UC and CD patients while positive correlations were observed between serum hepcidin and ferritin. However, such associations did not exist between serum hepcidin and folate and vitamin B12, indicating that hepcidin aggravates the severity of anemia in IBD patients by spoiling the balance of iron metabolism.

Alternatively, the relapsing and remitting inflammation are the clinical features of IBD. Recent studies [38, 39] have revealed that the expression of hepcidin is upregulated in patients with IBD and that the increased expression of hepcidin mediates ACD in CD patients, which is relevant to the serum IL-6 level in the environment of inflammation, but the association between hepcidin and the disease activity and the specific mechanisms involved in anemia remain to be confirmed in IBD. Although previous report [27] has proved that IL-6 is the main factor stimulating the expression of hepcidin through the STAT3 signaling pathway in patients with septicemia and burns, the high level of hepcidin mRNA is also observed in IL-6-deficient mice with chronic inflammation [28]. In our work, the high level of hepcidin was positively correlated with IL-6 and other proinflammatory cytokines like IL-17 and TNF-*α* in IBD patients. These results provide us with the assumption in that there are one or more kinds of other inflammatory factors existing in the microenvironment which have the same compacts on hepcidin as IL-6. TNF-*α* is regarded as one of the most important proinflammatory cytokines in the pathogenesis of IBD, and the TNF-*α* signaling pathway is also identified to play a vital role in making pleiotropic proinflammatory influence like increasing angiogenesis, activation, and recruitment of macrophage and effective T cells, the direct injure of Paneth cells and intestinal epithelial cells. To date, the infusion of anti-TNF-*α* mAb (e.g., IFX) has been widely applied in patients with IBD and achieved an impressively curative effect in clinic [40]. Previous studies [24, 41] have reported that the IFX therapy could improve anemia in patients with IBD, which is associated with the downregulated expression of hepcidin, although there are a large proportion of patients, especially patients with UC, who are not able to respond to IFX therapy. Moreover, recent work by Atkinson et al. [23] has also demonstrated that IFX treatment could also improve anemia in children with CD and is associated with a decrease of hepcidin expression. In this study, we analyzed the change of hepcidin before and after IFX therapy in both IFX-responsive and IFX-unresponsive CD patients and found that serum levels of hepcidin were decreased after IFX infusion in CD patients of the responsive group while no significant changes were observed in CD patients who failed to IFX treatment. In agreement with previous findings, our data further identify the close connection between anti-TNF-*α* therapy and hepcidin regulation [37, 42]. To conduct further experiment in our study, LO2 and HepG2 cell lines were cultured *in vitro* and TNF-*α* or IL-6 was selected as proinflammatory stimulators; the expression of hepcidin was found to be significantly increased when stimulated with TNF-*α* and IL-6, respectively, compared with controls. Importantly, an increase of hepcidin expression was rescued with the treatment of anti-TNF-*α* antibody or inhibitor of caspase-3/8 or NF-*κ*B inhibitor. Therefore, a hypothesis could be envisaged that anemia could be aggravated by a high level of TNF-*α* via stimulating the expression of hepcidin, which could be eliminated by antagonizing TNF-*α* or blocking the caspase-3/8- and NF-*κ*B-dependent pathways, which are involved in the delivery of TNF-*α* signaling.

## 5. Conclusions

Our data demonstrate that hepcidin is highly increased in active IBD patients, which further aggravates the severity of anemia by spoiling the balance of iron metabolism. Importantly, our study has paved the way to better understanding of how TNF-*α* upregulates the expression of hepcidin in IBD patients. It not only explores relevant mechanisms but also emphasizes the importance of the application of anti-TNF-*α* therapy in the management of anemia of IBD.

## Figures and Tables

**Table 1:** The clinical characteristics of IBD patients and healthy volunteers.

	HC	UC (A/R)^b^	CD (A/R)^b^
Patients	22	22/11	25/41
Age, years (range)	39.75 ± 16.17 (15-67)	37.63 ± 14.49 (12-79)	42.37 ± 17.95 (11-89)
Gender (M/F)	13/9	18/15	34/32
Medical therapy received			
5-ASA		19	40
Immunosuppressants		14	26
Biologics		0	0
Disease extent^a^			
Proctosigmoiditis (E1)		10	
Left-sided colitis (E2)		9	
Extensive colitis (E3)		14	
Disease location^a^			
Ileum only (L1)			21
Colon only (L2)			18
Ileum and colon (L3)			16
Upper gastrointestinal tract (L4a)			11

^a^Montreal classification; ^b^A/R: active/remission.

**Table 2:** The clinical characteristics of active CD patients.

	Response group	Failure group
Patients	19	13
Age, years (range)	36.41 ± 12.17 (18-57)	39.25 ± 13.83 (20-61)
Gender (M/F)	9/10	5/8
Medical therapy received		
5-ASA	12	7
Immunosuppressants	7	6
Biologics	0	0
Disease location		
Ileum only (L1)	4	3
Colon only (L2)	8	6
Ileum and colon (L3)	2	1
Upper gastrointestinal tract (L4a)	5	3
Disease duration (months)	31.56 ± 25.97	36.71 ± 23.59
Smoking history (months)	67.62 ± 21.31	58.94 ± 19.83

**Table 3:** Prevalence of MCV, MCH, MCHC, ferritin, vitamin B12, and folic acid in IBD patients.

Group	MCV (FL)	MCH (pGHCT)	MCHC (g/L)	Ferritin (ng/mL)	Vitamin B12 (pmol/L)	Folic acid (ng/mL)
Anemia CD (*n* = 38)	80.78 ± 0.79	25.18 ± 0.32	309.4 ± 1.44	166.7 ± 24.15	470.1 ± 46.90	8.45 ± 0.58
No Anemia CD (*n* = 28)	87.32 ± 0.51^∗^	29.20 ± 0.23^∗^	333.2 ± 1.32^∗^	158.8 ± 31.48	476.6 ± 47.26	8.19 ± 1.05
Anemia UC (*n* = 21)	84.50 ± 1.00	26.60 ± 0.42	313.1 ± 1.79	259.7 ± 47.98	506.4 ± 40.24	6.69 ± 0.77
No Anemia UC (*n* = 12)	87.98 ± 0.45^^^	29.15 ± 0.21^∗^	331.7 ± 0.88^∗^	247.5 ± 33.29	500.6 ± 30.63	7.08 ± 0.73

^∗^ *P* < 0.0001 vs. the anemia group; ^^^*P* < 0.0005 vs. the anemia group.

## Data Availability

All data generated or analyzed during this study are included in this article.

## Abbreviations

CD: Crohn's disease
DC: Dendritic cell
IBD: Inflammatory bowel disease
IEC: Intestinal epithelial cell
IFN-*γ*: Interferon *γ*
IL: Interleukin
LP: Lamina propria
PBMC: Peripheral blood mononuclear cell
TNF-*α*: Tumor necrosis factor-*α*
UC: Ulcerative colitis
CDAI: Crohn's disease activity index.

## Supplementary Materials

Supplementary Materials Supplementary Figure 1 no correlation exists between the levels of hepcidin and folic acid and vitamin B12 in the sera of IBD patients. (a) Correlation between the levels of hepcidin and folic acid in UC patients (*n* = 30), (b) correlation between the levels of hepcidin and folic acid in CD patients (*n* = 66), (c) correlation between the levels of hepcidin and vitamin B12 in UC patients (*n* = 30), and (d) correlation between the levels of hepcidin and vitamin B12 in CD patients (*n* = 66). Spearman's correlation analysis was used for correlation analysis. *r* and *P* values are shown in each panel. Supplementary Figure 2: no significant differences in the viability of LO2 cells and HepG2 cells after exposure to TNF-*α*, IFX, and JNK-IN-8, BAY 11-7802, and Z-DEVD-FMK, respectively, for 12 h. (a) LO2 cell line and (b) HepG2 cell line were incubated with TNF-*α* (10 ng/mL) in the presence of anti-TNF-*α* mAb (IFX, 50 ng/*μ*L), JNK inhibitor (JNK-IN-8, 10 *μ*M), NF-*κ*B inhibitor (BAY 11-7082, 10 *μ*M), and caspase-3/8 inhibitor (Z-DEVD-FMK, 50 *μ*M), respectively, as indicated. One-way ANOVA was performed for statistical analysis.

## References

[1] Prochaska M. T., Newcomb R., Block G., Park B., Meltzer D. O. (2017). Association between anemia and fatigue in hospitalized patients: does the measure of anemia matter. *Journal of Hospital Medicine*.

[2] Jonefjall B., Simren M., Lasson A., Öhman L., Strid H. (2018). Psychological distress, iron deficiency, active disease and female gender are independent risk factors for fatigue in patients with ulcerative colitis. *United European Gastroenterology Journal*.

[3] Nielsen O. H., Soendergaard C., Vikner M. E., Weiss G. (2018). Rational Management of iron-deficiency anaemia in inflammatory bowel disease. *Nutrient*.

[4] Vagianos K., Clara I., Carr R. (2016). What Are Adults With Inflammatory Bowel Disease (IBD) Eating? A Closer Look at the Dietary Habits of a Population‐Based Canadian IBD Cohort. *JPEN Journal of Parenteral and Enteral Nutrition*.

[5] Theurl I., Aigner E., Theurl M. (2009). Regulation of iron homeostasis in anemia of chronic disease and iron deficiency anemia: diagnostic and therapeutic implications. *Blood*.

[6] Horina J. H., Petritsch W., Schmid C. R. (1993). Treatment of anemia in inflammatory bowel disease with recombinant human erythropoietin: results in three patients. *Gastroenterology*.

[7] Semrin G., Fishman D. S., Bousvaros A. (2006). Impaired intestinal iron absorption in Crohn’s disease correlates with disease activity and markers of inflammation. *Inflammatory Bowel Diseases*.

[8] Gerasimidis K., Barclay A., Papangelou A. (2013). The epidemiology of anemia in pediatric inflammatory bowel disease: prevalence and associated factors at diagnosis and follow-up and the impact of exclusive enteral nutrition. *Inflammatory Bowel Diseases*.

[9] Filmann N., Rey J., Schneeweiss S. (2014). Prevalence of anemia in inflammatory bowel diseases in European countries: a systematic review and individual patient data meta-analysis. *Inflammatory Bowel Diseases*.

[10] Hirono I., Hwang J. Y., Ono Y. (2005). Two different types of hepcidins from the Japanese flounder Paralichthys olivaceus. *The FEBS Journal*.

[11] Tian L., Chen S., Liu H. (2016). In vivo effects of Pichia pastoris-expressed antimicrobial peptide hepcidin on the community composition and metabolism gut microbiota of rats. *PLoS One*.

[12] Falzacappa M. V. V., Muckenthaler M. U. (2005). Hepcidin: iron-hormone and anti-microbial peptide. *Gene*.

[13] Tavanti A., Maisetta G., Gaudio D. G. (2011). Fungicidal activity of the human peptide hepcidin 20 alone or in combination with other antifungals against _Candida glabrata_ isolates. *Peptides*.

[14] Park C. H., Valore E. V., Waring A. J., Ganz T. (2001). Hepcidin, a urinary antimicrobial peptide synthesized in the liver. *The Journal of Biological Chemistry*.

[15] Ramey G., Deschemin J. C., Durel B., Canonne-Hergaux F., Nicolas G., Vaulont S. (2010). Hepcidin targets ferroportin for degradation in hepatocytes. *Haematologica*.

[16] Donovan A., Lima C. A., Pinkus J. L. (2005). The iron exporter ferroportin/Slc40a1 is essential for iron homeostasis. *Cell Metabolism*.

[17] Frazer D. M., Darshan D., Anderson G. J. (2011). Intestinal iron absorption during suckling in mammals. *Biometals*.

[18] Sakamori R., Takehara T., Tatsumi T. (2010). STAT3 signaling within hepatocytes is required for anemia of inflammation in vivo. *Journal of Gastroenterology*.

[19] Lee P., Peng H., Gelbart T., Beutler E. (2004). The IL-6- and lipopolysaccharide-induced transcription of hepcidin in HFE-, transferrin receptor 2-, and beta 2-microglobulin-deficient hepatocytes. *Proceedings of the National Academy of Sciences of the United States of America*.

[20] Deora A., Hegde S., Lee J. (2017). Transmembrane TNF-dependent uptake of anti-TNF antibodies. *MAbs*.

[21] Song S.-N. J., Iwahashi M., Tomosugi N. (2013). Comparative evaluation of the effects of treatment with tocilizumab and TNF-*α* inhibitors on serum hepcidin, anemia response and disease activity in rheumatoid arthritis patients. *Arthritis Research & Therapy*.

[22] Wu W., Song Y., He C. (2015). Divalent metal-ion transporter 1 is decreased in intestinal epithelial cells and contributes to the anemia in inflammatory bowel disease. *Scientific Reports*.

[23] Atkinson M. A., Leonard M. B., Herskovitz R., Baldassano R. N., Denburg M. R. (2018). Changes in hepcidin and hemoglobin after anti-TNF-alpha therapy in children and adolescents with Crohn disease. *Journal of Pediatric Gastroenterology and Nutrition*.

[24] Cavallaro F., Duca L., Pisani L. F. (2017). Anti-TNF-mediated modulation of prohepcidin improves iron availability in inflammatory bowel disease, in an IL-6-mediated fashion. *Canadian Journal of Gastroenterology and Hepatology*.

[25] Liu C., Xia X., Wu W. (2013). Anti-tumour necrosis factor therapy enhances mucosal healing through down-regulation of interleukin-21 expression and T helper type 17 cell infiltration in Crohn's disease. *Clinical and Experimental Immunology*.

[26] Munoz M., Garcia-Erce J. A., Remacha A. F. (2011). Disorders of iron metabolism. Part II: iron deficiency and iron overload. *Journal of Clinical Pathology*.

[27] Xin H., Wang M., Tang W. (2016). Hydrogen sulfide attenuates inflammatory hepcidin by reducing IL-6 secretion and promoting SIRT1-mediated STAT3 deacetylation. *Antioxidants & Redox Signaling*.

[28] Gardenghi S., Renaud T. M., Meloni A. (2014). Distinct roles for hepcidin and interleukin-6 in the recovery from anemia in mice injected with heat-killed Brucella abortus. *Blood*.

[29] Koutroubakis I. E., Ramos-Rivers C., Regueiro M. (2015). The influence of anti-tumor necrosis factor agents on hemoglobin levels of patients with inflammatory bowel disease. *Inflammatory Bowel Diseases*.

[30] Guagnozzi D., Lucendo A. J. (2014). Anemia in inflammatory bowel disease: a neglected issue with relevant effects. *World Journal of Gastroenterology*.

[31] Wang X., Wu Z., Chen Y. (2015). Increased prevalence and incidence of anemia among adults in transforming rural China: two cross-sectional surveys. *BMC Public Health*.

[32] Li J., Zhang Q., Fan X. (2017). The long noncoding RNA TUG1 acts as a competing endogenous RNA to regulate the Hedgehog pathway by targeting miR-132 in hepatocellular carcinoma. *Oncotarget*.

[33] Galesloot T. E., Vermeulen S. H., Geurts-Moespot A. J. (2011). Serum hepcidin: reference ranges and biochemical correlates in the general population. *Blood*.

[34] Verma S., Cherayil B. J. (2017). Iron and inflammation - the gut reaction. *Metallomics*.

[35] Weiss G. (2002). Pathogenesis and treatment of anaemia of chronic disease. *Blood Rev*.

[36] Arabul M., Celik M., Aslan O. (2013). Hepcidin as a predictor of disease severity in acute pancreatitis: a single center prospective study. *Hepato-Gastroenterology*.

[37] Abdel-Khalek M. A., El-Barbary A. M., Essa S. A., Ghobashi A. S. (2011). Serum hepcidin: a direct link between anemia of inflammation and coronary artery atherosclerosis in patients with rheumatoid arthritis. *The Journal of Rheumatology*.

[38] Basseri R. J., Nemeth E., Vassilaki M. E. (2013). Hepcidin is a key mediator of anemia of inflammation in Crohn’s disease. *Journal of Crohn's & Colitis*.

[39] Bergamaschi G., Di Sabatino A., Albertini R. (2013). Serum hepcidin in inflammatory bowel Diseases. *Inflammatory Bowel Diseases*.

[40] Nishida Y., Hosomi S., Watanabe K. (2018). Serum interleukin-6 level is associated with response to infliximab in ulcerative colitis. *Scandinavian Journal of Gastroenterology*.

[41] Karaskova E., Volejnikova J., Holub D. (2019). Changes in serum hepcidin levels in children with inflammatory bowel disease during anti‐inflammatory treatment. *Journal of Paediatrics and Child Health*.

[42] Doyle M. K., Rahman M. U., Frederick B. (2013). Effects of subcutaneous and intravenous golimumab on inflammatory biomarkers in patients with rheumatoid arthritis: results of a phase 1, randomized, open-label trial. *Rheumatology (Oxford)*.

