# Supplementary material for: Anti-TNF-α Monoclonal Antibody Therapy Improves Anemia through Downregulating Hepatocyte Hepcidin Expression in Inflammatory Bowel Disease
Source: Mediators Inflamm. 2019 Nov 13;2019:4038619. doi: 10.1155/2019/4038619 (PMC6878771; doi:10.1155/2019/4038619)
Supplement: Supplementary Materials — Supplementary Figure 1 no correlation exists between the levels of hepcidin and folic acid and vitamin B12 in the sera of IBD patients. (a) Correlation between the levels of hepcidin and folic acid in UC patients (n = 30), (b) correlation between the levels of hepcidin and folic acid in CD patients (n = 66), (c) correlation between the levels of hepcidin and vitamin B12 in UC patients (n = 30), and (d) correlation between the levels of hepcidin and vitamin B12 in CD patients (n = 66). Spearman's correlation analysis was used for correlation analysis. r and P values are shown in each panel. Supplementary Figure 2: no significant differences in the viability of LO2 cells and HepG2 cells after exposure to TNF-α, IFX, and JNK-IN-8, BAY 11-7802, and Z-DEVD-FMK, respectively, for 12 h. (a) LO2 cell line and (b) HepG2 cell line were incubated with TNF-α (10 ng/mL) in the presence of anti-TNF-α mAb (IFX, 50 ng/μL), JNK inhibitor (JNK-IN-8, 10 μM), NF-κB inhibitor (BAY 11-7082, 10 μM), and caspase-3/8 inhibitor (Z-DEVD-FMK, 50 μM), respectively, as indicated. One-way ANOVA was performed for statistical analysis. [file 4038619.f1.pptx]

## Slide 1
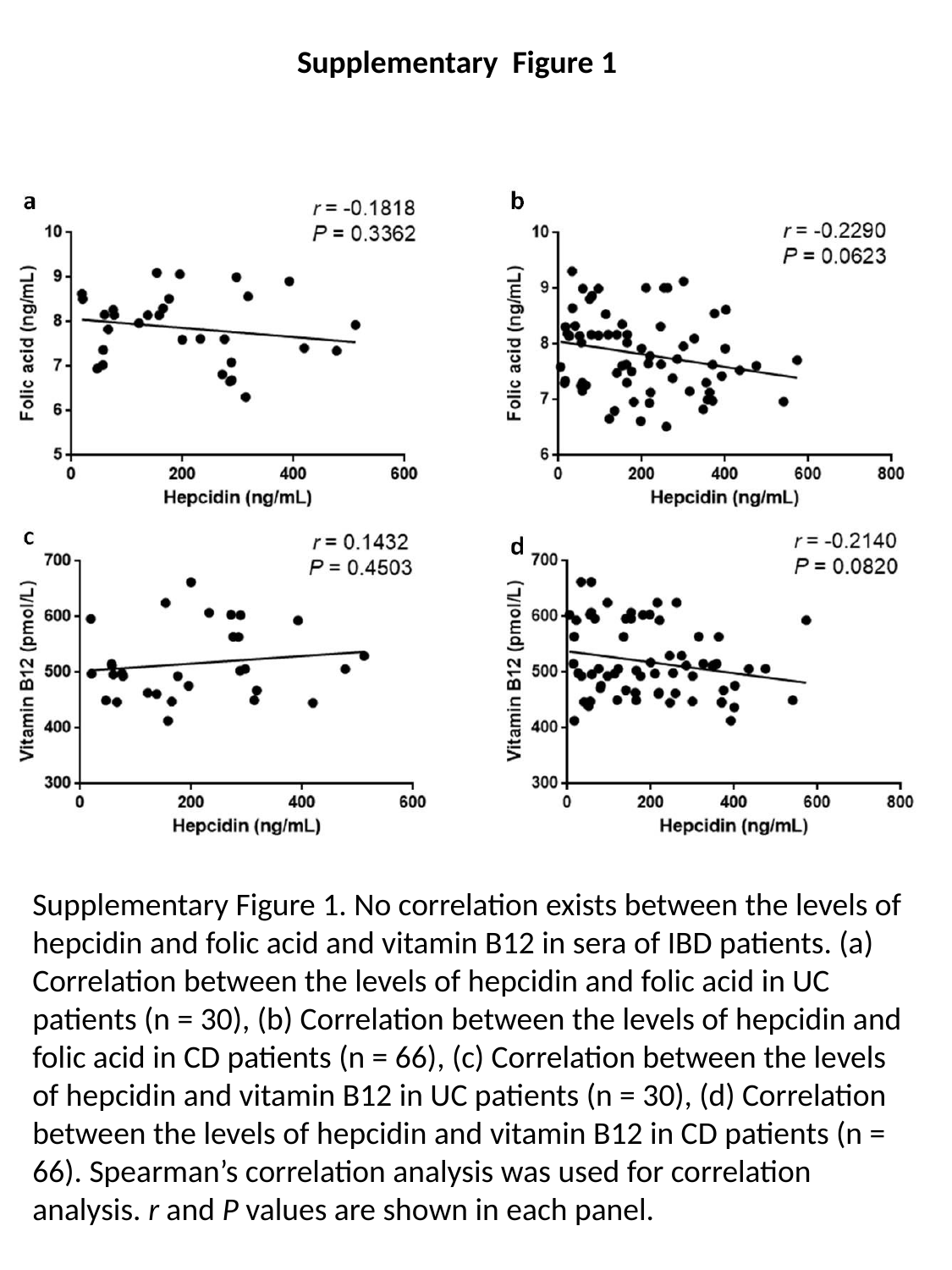

Supplementary Figure 1
Supplementary Figure 1. No correlation exists between the levels of hepcidin and folic acid and vitamin B12 in sera of IBD patients. (a) Correlation between the levels of hepcidin and folic acid in UC patients (n = 30), (b) Correlation between the levels of hepcidin and folic acid in CD patients (n = 66), (c) Correlation between the levels of hepcidin and vitamin B12 in UC patients (n = 30), (d) Correlation between the levels of hepcidin and vitamin B12 in CD patients (n = 66). Spearman’s correlation analysis was used for correlation analysis. r and P values are shown in each panel.

## Slide 2
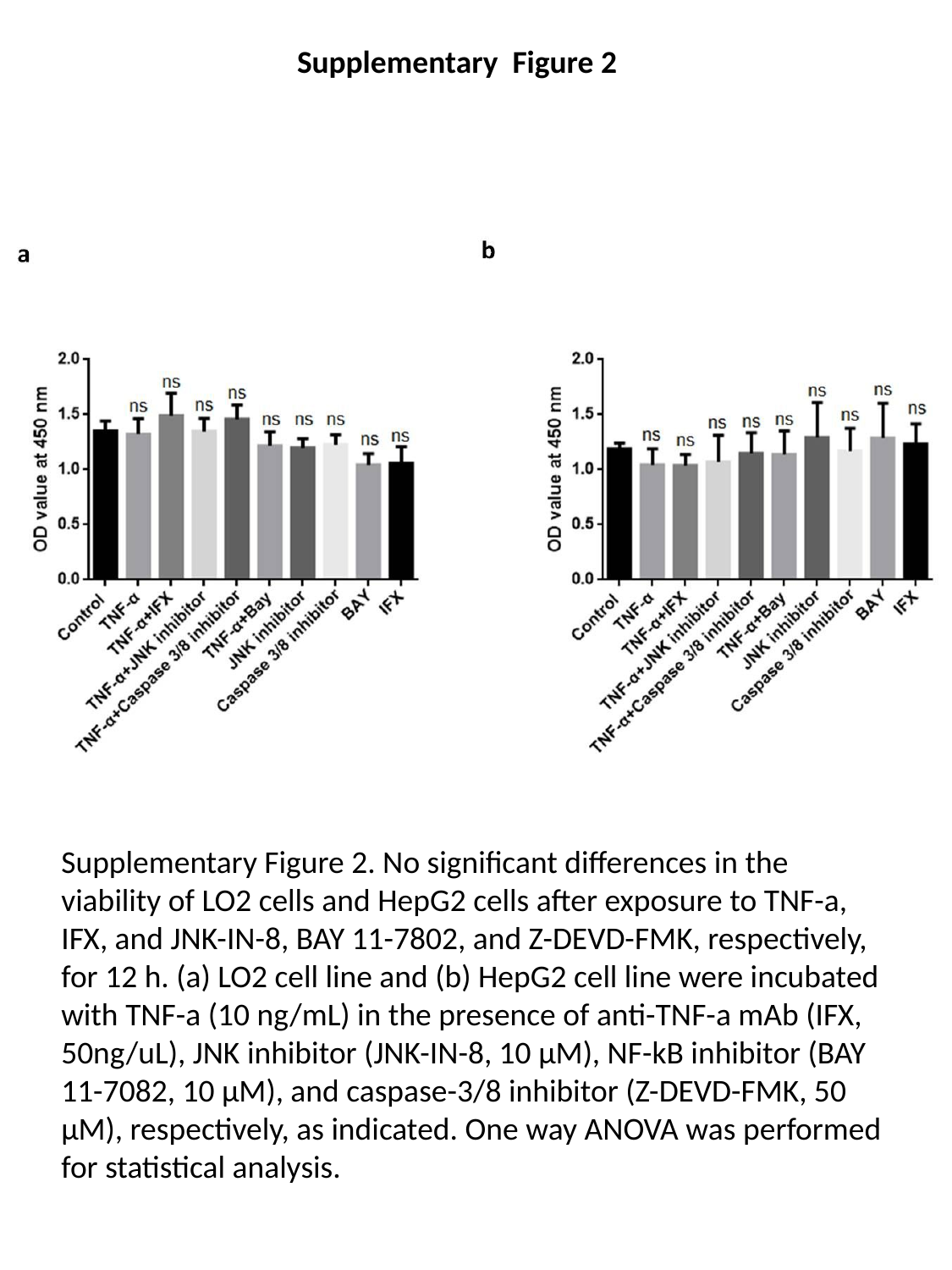

Supplementary Figure 2
Supplementary Figure 2. No significant differences in the viability of LO2 cells and HepG2 cells after exposure to TNF-a, IFX, and JNK-IN-8, BAY 11-7802, and Z-DEVD-FMK, respectively, for 12 h. (a) LO2 cell line and (b) HepG2 cell line were incubated with TNF-a (10 ng/mL) in the presence of anti-TNF-a mAb (IFX, 50ng/uL), JNK inhibitor (JNK-IN-8, 10 μM), NF-kB inhibitor (BAY 11-7082, 10 μM), and caspase-3/8 inhibitor (Z-DEVD-FMK, 50 μM), respectively, as indicated. One way ANOVA was performed for statistical analysis.
